# Identification of Epithelial–Mesenchymal Transition-Related Prognostic lncRNAs Biomarkers Associated With Melanoma Microenvironment

**DOI:** 10.3389/fcell.2021.679133

**Published:** 2021-05-31

**Authors:** Bo Xiao, Liyan Liu, Zhuoyuan Chen, Aoyu Li, Pingxiao Wang, Cheng Xiang, Yi Zeng, Hui Li, Tao Xiao

**Affiliations:** ^1^Department of Orthopedics, Second Xiangya Hospital, Central South University, Changsha, China; ^2^Orthopedic Biomedical Materials Engineering Laboratory of Hunan Province, Changsha, China

**Keywords:** melanoma, epithelial to mesenchymal transition, lncRNA, prognostic, tumor microenvironment

## Abstract

Melanoma is the most common cancer of the skin, associated with a worse prognosis and distant metastasis. Epithelial–mesenchymal transition (EMT) is a reversible cellular biological process that plays significant roles in diverse tumor functions, and it is modulated by specific genes and transcription factors. The relevance of EMT-related lncRNAs in melanoma has not been determined. Therefore, RNA expression data and clinical features were collected from the TCGA database (*N* = 447). Melanoma samples were randomly assigned into the training (315) and testing sets (132). An EMT-related lncRNA signature was constructed via comprehensive analyses of lncRNA expression level and corresponding clinical data. The Kaplan-Meier analysis showed significant differences in overall survival in patients with melanoma in the low and high-risk groups in two sets. Receiver operating characteristic (ROC) curves were used to measure the performance of the model. Cox regression analysis indicated that the risk score was an independent prognostic factor in two sets. Besides, a nomogram was constructed based on the independent variables. Gene Set Enrichment Analysis (GSEA) was applied to evaluate the potential biological functions in the two risk groups. Furthermore, the melanoma microenvironment was evaluated using ESTIMATE and CIBERSORT algorithms in the risk groups. This study indicates that EMT-related lncRNAs can function as potential independent prognostic biomarkers for melanoma survival.

## Introduction

Melanoma is the fifth and seventh most common cancer in males and females, respectively (Wu et al., 2020). Currently, with a growth rate of 3–5% per year, there are an estimated 20,000 new cases of melanoma each year (Li and Chen, 2013). Numerous treatment methods such as surgery, chemotherapy, radiotherapy, immunotherapy, and targeted therapy are used in the treatment of localized tumors. Surgery remains the main treatment option for melanoma with 90% cure rates. However, for advanced tumors, the mortality rate of melanoma patients is high (Mishra et al., 2018). The recent development of immunotherapy has greatly improved the treatment of melanoma. However, the prognosis of patients with melanoma remains poor, mainly due to resistance to chemotherapy and/or radiotherapy, aggressive clinical behavior, and higher metastatic potential, resulting in high mortality rates (Bommareddy et al., 2017). Thus, there is an urgent need to identify novel biomarkers or therapeutic targets for predicting melanoma progression, prognosis, and treatment effects.

Epithelial-mesenchymal transition (EMT) is a cellular biological process by which epithelial cells lose their cell-cell adhesion and polarity features and gain the migratory and invasive features to become mesenchymal cells (Pastushenko and Blanpain, 2019; Bakir et al., 2020). Studies have shown that EMT plays diverse roles in tumor functions, including tumorigenesis, tumor progression, metastasis, migration, tumor stemness, intravasation, and chemotherapy resistance (Brabletz, 2012; De Craene and Berx, 2013; Nieto et al., 2016). As an EMT-related gene, RhoC is a member of the Ras-homologous (Rho) GTPase family, has been reported to be associated with worse disease-free survival and overall survival in melanoma patients (Boone et al., 2009). Besides, the overexpression of coxsackie and adenovirus receptor (CXADR) is not only associated with worse survival of melanoma patients but also enhances tumor progression via EMT (Wenandy et al., 2008). Overexpression of CXADR seems to resistant breast cancer cells to TGFβ-induced EMT and its expression associates with overall survival (Nilchian et al., 2019). Besides, EMT may promote tumor growth and metastasis not only through a direct reordering of cancer cells but also through reprogramming of the immune response in the tumor microenvironment (Lou et al., 2016). Thus, the EMT-related genes/lncRNAs may act as potential targets for future melanoma treatment. However, the relationship between the expression of EMT-related lncRNA, their prognostic value, and the pathological characteristics of melanoma has not been investigated.

In this study, we constructed a prognostic model using lncRNA expression data and relevant clinical data of melanoma obtained from the TCGA database. To build a prognostic model, we relied on the expression of EMT-related lncRNA, and we analyzed the correlation between the subgroups and the tumor microenvironment.

## Materials and Methods

### Public Data Sources

FPKM lncRNA expression data and relevant clinical of melanoma patients were downloaded from The Cancer Genome Atlas (TCGA). The merge script by Perl and Ensembl database were used to combine RNA-seq results into a matrix of gene symbols. The melanoma dataset contained 471 melanoma tissues and 1 adjacent normal tissue. To enhance the quality analysis, cases without complete survival data as well as those whose overall survival (OS) was less than 30 days were excluded from the study. A total of 447 participants were included in the analysis.

### Correlation Analysis

A total of 200 EMT-related genes were downloaded from the MSigDB v7.2 (Molecular Signature Database). Pearson’s correlation analysis was applied to determine the relationship between expressed EMT-related genes and the expression of lncRNAs in melanoma patient samples (correlation coefficient > 0.35, *p* < 0.001). We then randomly divided the melanoma samples into the training and testing cohorts using the R package “caret.” The expression levels of EMT-related lncRNAs in the training set were further used to construct the prognostic model.

### Construction and Validation of the Prognostic Signature

Univariate Cox regression analysis was performed using the “survival” package in R to determine the EMT-related lncRNAs associated with the overall survival of melanoma patients in the training set (*p* < 0.01). LASSO regression analysis was performed using the “glmnet” package in R to determine the most significant lncRNAs. We built a risk signature through multivariate Cox regression using the “survminer” package in R. The formula used was: risk score = Σ_*i*_ coefficient (lncRNAi) × expression (lncRNAi). Thus, the samples in the training and testing sets were classified as low vs. high groups based on the median risk score. The Kaplan-Meier survival curves were drawn using the “survival” package. Moreover, time-dependent receiver-operating characteristic (ROC) was used to examine the prognostic value of the risk signature using the “survivalROC” package.

### Evaluation of the Independent Prognostic Value of the Risk Score

Univariate and multivariate Cox regression analyses were performed based on the risk score and available clinical data in the training and testing sets to verify whether the risk score is an independent prognostic predictor.

### Construction and Verification of the Nomogram

The nomogram was constructed from the 1-, 3-, and 5-year overall survival data of melanoma patients and the independent prognostic factors using the “rms” R package. The 3-, 5-year calibration plots were used to evaluate the accuracy of the nomogram.

### GSEA Enrichment Analysis

GSEA (Gene Set Enrichment Analysis) was employed to identify the biological functions and pathways associated with the high and low-risk groups. FDR < 5% and *P* < 0.05 were considered to be statistically significant.

### Evaluation of Tumor Microenvironment

ESTIMATE was designed to calculate the immune and stromal scores within the tumor microenvironment based on the expression of immune and stromal cells (Yoshihara et al., 2013). ESTIMATE algorithm was used to calculate the immune score, stromal score, ESTIMATE score, and tumor purity and also analyze the association between the risk groups and tumor microenvironment. Kaplan-Meier method was applied to determine the relationship between the scores and overall survival of melanoma patients.

### Evaluation of Immune Cells Infiltration

To further explore the difference between immune cell infiltration in the low and high-risk groups, the CIBERSORT algorithm was used to determine the fraction of 22 immune cells from gene expression data in melanoma cases (Newman et al., 2015). Samples with a CIBERSORT output of *P*-value less than 0.05 were used for further analyses. We used the Wilcoxon rank-sum test to identify the fraction of immune cells that had remarkable differences in the proportion between the two risk groups (high vs. low). Kaplan–Meier method was used to determine the relationship between the 22 infiltrating immune cells and OS of melanoma patients. Besides, Pearson correlation coefficient was applied to evaluate the correlation between risk score and immune cells.

### Statistical Analysis

All statistical data were analyzed by R (version 4.0.2) and Perl. Chi-square or fisher test to analyze categorical variables, whereas Wilcoxon test was used to analyze the continuous data. The difference in survival was computed utilizing the K–M and the log-rank test methods. *P* < 0.05 was considered statistically significant.

## Results

### EMT-Associated lncRNA

A total of 200 EMT-related genes were obtained from the MSigDB database (M340) (Supplementary Table 1). We distinguished EMT-related lncRNAs via correlation analysis. Based on the criteria, we identified 667 EMT-related lncRNAs. The expression level of 667 EMT-related lncRNAs and the clinical information of the 447 melanoma samples were used for further analysis. The 447 melanoma patients were randomly assigned into the training (315) and testing sets (132) using the “caret” package.

### Construction and Verification of the EMT-Related lncRNA Signature

**FIGURE 1:**
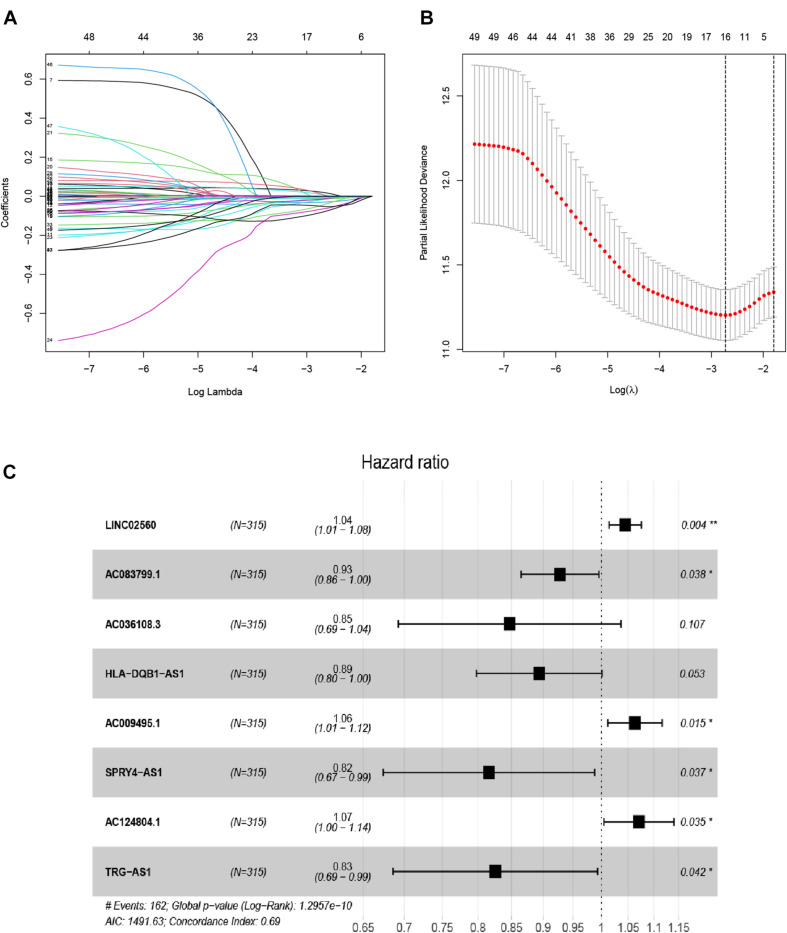
Distinction of EMT-related lncRNAs correlated with melanoma prognosis: LASSO **(A,B)**, and multivariate Cox regression analysis **(C)**.

Here, 315 melanoma samples with overall survival of over 1 month, and 667 EMT-related lncRNAs from TCGA-SCKM were used to construct the prognostic model. Firstly, univariate Cox regression analysis revealed that a total of 49 EMT-related lncRNAs had a significant association with overall survival (Supplementary Table 2) in the training set. Then, the LASSO regression analysis of the 49 EMT-related lncRNAs identified 16 EMT-related lncRNAs which were highly associated with OS (Figures 1A,B). Finally, from these 16 EMT-related lncRNAs, 8 EMT-related lncRNAs were determined utilizing the multivariate Cox regression analysis and employed to establish an eight EMT-related lncRNAs signature model, and among them, three lncRNAs (LINC02560, AC009495.1, and AC124804.1) were associated with increased risk with HRs > 1, while the other five lncRNAs (AC083799.1, AC036108.3, HLA-DQB1-AS1, SPRY4-AS1, and TRG-AS1) were protective lncRNAs with HRs < 1 (Supplementary Table 3 and Figure 1C). The risk score was established using the following formula: Risk score = (0.04325378^∗^expression of LINC02560) + (−0.0747787^∗^ expression of AC083799.1) + (−0.1658322^∗^expression of AC036108.3) + (−0.1121718^∗^ expression of HLA-DQB1-AS1) + (0.06087171^∗^expression of AC009495.1) + (−0.2031776^∗^ expression of SPRY4-AS1) + (0.0681405^∗^expression of AC124804.1) + (−0.1917719^∗^expression of TRG-AS1).

**FIGURE 2:**
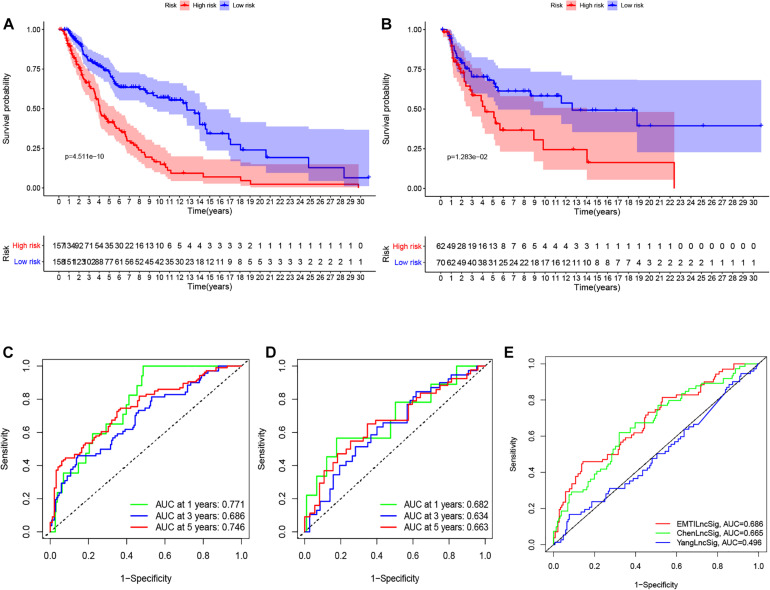
K–M survival analysis **(A,B)**, ROC curves **(C,D)** at 1-, 3-, and 5-years post diagnosis of immune-related lncRNA signature in melanoma in training and testing cohort. The ROC curve at 3 years of overall survival for the EMTLncSig, ChenLncSig and YangLncSig **(E)**.

**FIGURE 3:**
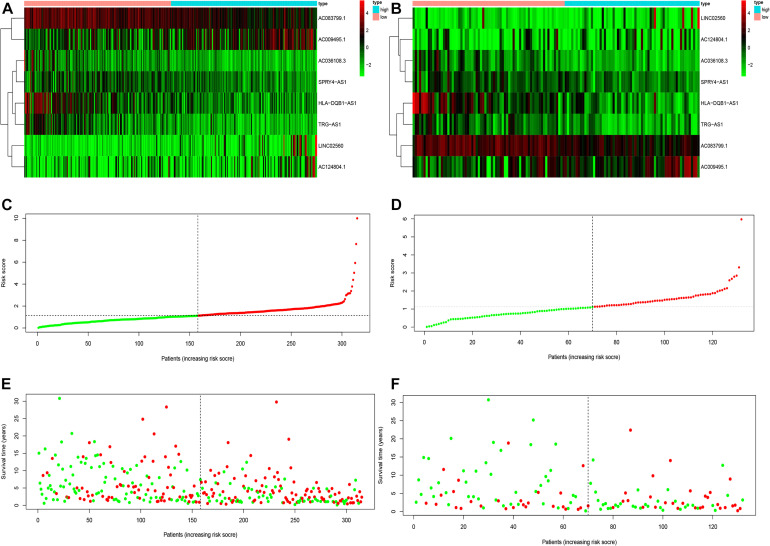
The heatmaps **(A,B)**, risk score **(C,D)**, and survival status analysis **(E,F)** of immune-related lncRNA signature in melanoma in training and testing cohort.

To determine the potential prognostic value of the EMT-related lncRNA signature in predicting the OS of melanoma patients, we divided the patients into two risk groups (high vs. low) based on the median risk score. The Kaplan-Meier method was used to evaluate the difference in OS between the two risk groups. As shown in Figures 2A,B, patients in the low-risk group had better survival compared with patients in the high-risk group in both the training and testing cohorts. The 5-year survival rate in the low-risk group was 72.32%, while that in the high-risk group was 40.11%. ROC curves were used to demonstrate the precision of the eight EMT-related lncRNAs in predicting the 1-, 3-, and 5-year OS of melanoma patients. The 1-, 3-, and 5-year survival AUC values were 0.771, 0.686, and 0.746 in the training set, and 0.682, 0.634, and 0.663 in the testing set (Figures 2C,D). Additionally, Figures 3A–F displays the heatmap and status of the training and testing set showing the melanoma samples in the high-risk group associated with more deaths, and the expression of LINC02560, AC009495.1, and AC124804.1. However, the low-risk group highly expressed AC083799.1, AC036108.3, HLA-DQB1-AS1, SPRY4-AS1, and TRG-AS1.

### Performance Comparison of EMTLncSig With Other lncRNA Signatures

We compared the prediction value of the EMT-related lncRNA signature with two published lncRNA signatures: one lncRNA signature derived from Chen’s study and another lncRNA signature derived from Yang’s study, and both studies utilized the same data from the TCGA database. The AUC value at 3 years for the EMT-related lncRNA signature was 0.686, which was higher than that reported by ChenlncSig (0.665) and YanglncSig (0.496) (Figure 2E). This illustrates the good prognostic performance of the EMT-related lncRNA signature in predicting survival compared with the two previously reported signatures.

### Identification of Independent Prognostic Factors

**FIGURE 4:**
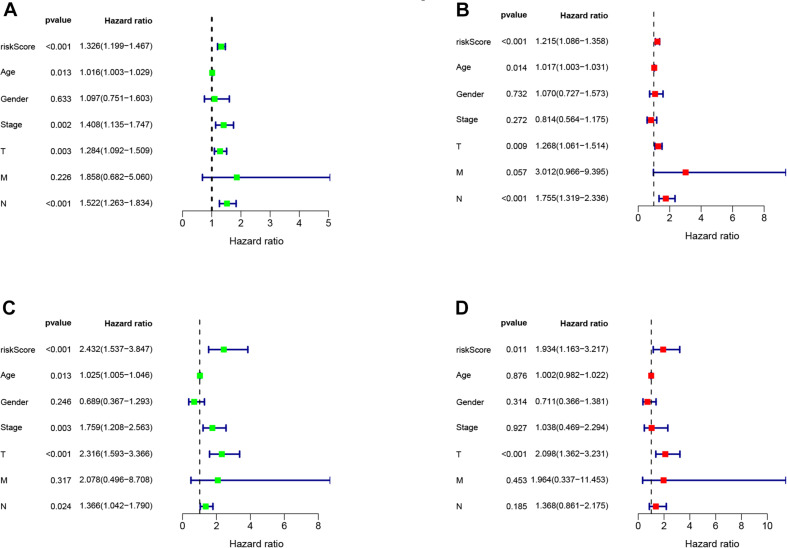
The Cox regression analysis of risk score, age, gender, T, N, M, and tumor stage in training **(A,B)**, testing **(C,D)** cohort.

**FIGURE 5:**
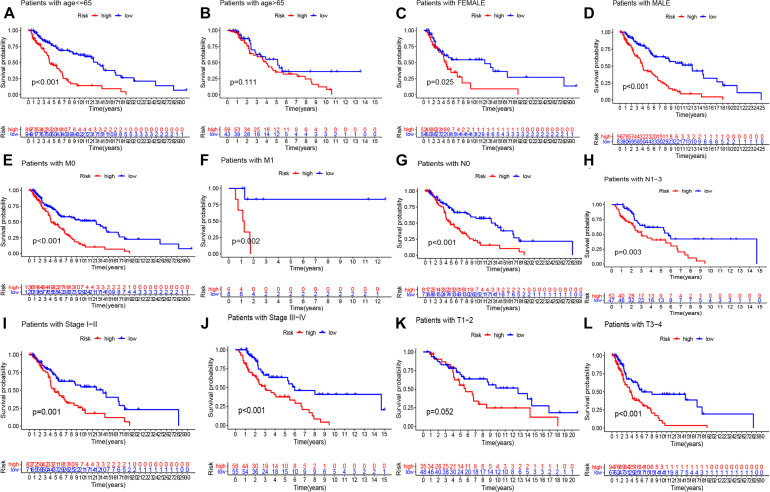
K–M methods for the two risk group (high vs. low) categorized by clinical variables, comprising age **(A,B)**, gender **(C,D)**, M **(E,F)**, N **(G,H)**, stage **(I,J)**, and T **(K,L)**.

We conducted univariate and multivariate Cox regression analysis to determine if the risk score can be considered as an independent prognostic predictor of overall survival in melanoma patients. The univariate Cox regression analysis demonstrated that the risk score was an independent variable for forecasting the prognosis of melanoma patients in the training (*P* < 0.001, HR = 1.326, 95% CI: 1.199–1.467 and *P* < 0.001, Figure 4A) and testing (*P* < 0.001, HR = 1.326, 95% CI: 1.199–1.467 and *P* < 0.001, HR: 2.432, 95% CI: 1.537–3.847, Figure 4C) sets. The multivariate Cox regression analysis also illustrated that the risk score was an independent prognostic factor for melanoma patients in both training (*P* < 0.001, HR = 1.215, 95% CI: 1.086–1.358, Figure 4B) and testing (*P* = 0.011, HR = 1.934, 95% CI: 1.163–3.217, Figure 4D) sets. Besides, to investigate the prognostic value of the signature in melanoma patients classified by clinical factors, we divided the samples into different groups according to age, gender, stage, T, N, and M. As displayed in Figure 5, the OS of melanoma patients in the high-risk group was significantly poorer than that of the low-risk group (*P* < 0.05). Both outcomes showed that EMT-related lncRNA signature had the potential to predict the overall survival of melanoma patients.

### Construction and Validation of the Nomogram

**FIGURE 6:**
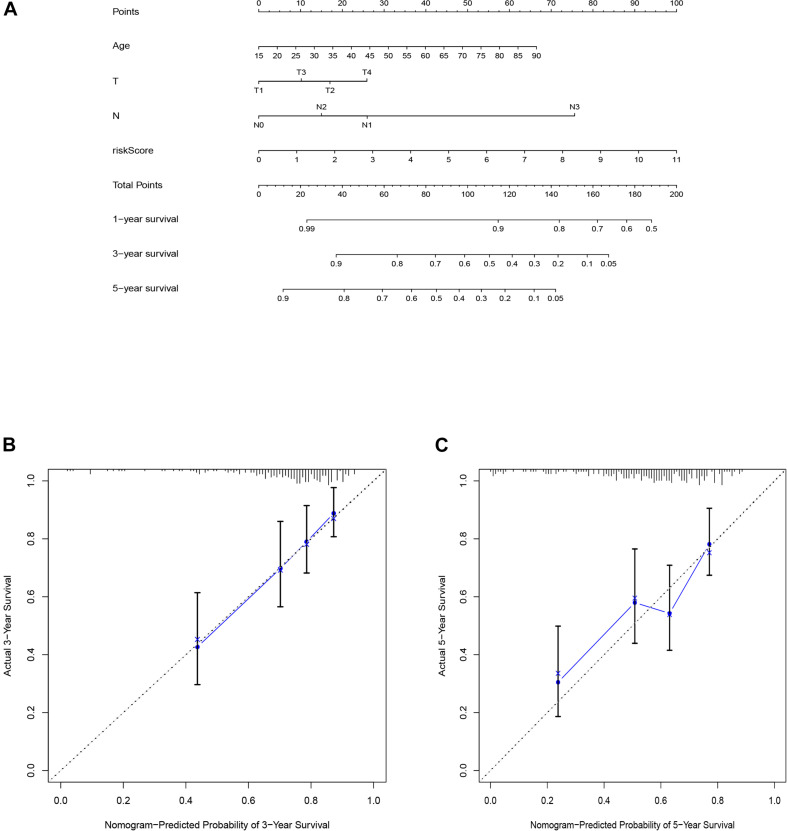
The nomogram of training set applied to predict the survival **(A)**. Calibration plots used to predict the 3-**(B)**, and 5-year survival **(C)**.

To predict the 1-, 3-, and 5-years overall survival of the patients, we established the nomogram using the risk score and other independent clinical variables in the training set (Figure 6A). The calibration curve displayed good performance for predicting the 3- and 5-years OS in melanoma patient samples (Figures 6B,C).

### Identification of GSEA-Derived EMT-Related lncRNA Signature

**FIGURE 7:**
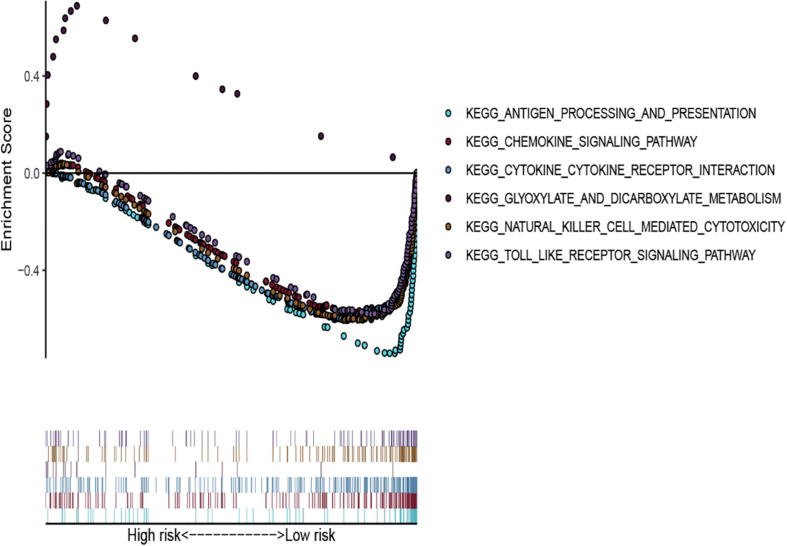
The GSEA for EMT-related lncRNA signature in the whole cohort.

We used GSEA to analyze the different pathways that were significantly enriched between the two risk groups (low vs. high). Samples in the high-risk group were enriched in glyoxylate and dicarboxylate metabolism, while samples in the low-risk group were enriched in the toll-like receptor signaling pathway, chemokine signaling pathway, natural killer cell-mediated cytotoxicity, cytokine-cytokine receptor interaction, and antigen processing and presentation (Figure 7).

### The Relationship Between Tumor Microenvironment and Risk Groups

**FIGURE 8:**
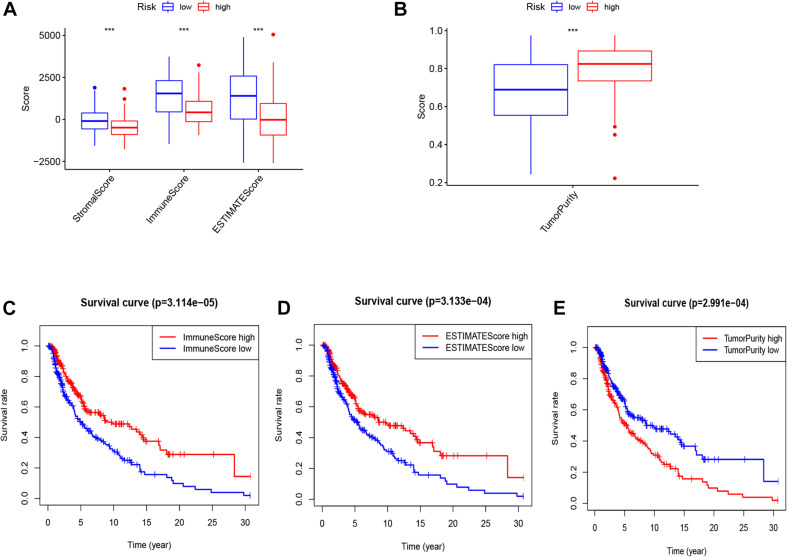
The score of stromal, immune, ESTIMATE **(A)**, and tumor purity **(B)** in two risk groups (high vs. low). K–M curve of OS between high and low immune, ESTIMATE and tumor purity **(C–E)**.

As displayed in Figures 8A,B, patients in the low-risk group had a significantly higher stromal score, immune score, and ESTIMATE score; and a remarkably lower tumor purity score in the whole (training and testing) set. Moreover, patients with high ESTIMATE scores and/or immune scores were associated with a higher survival rate, while those with higher tumor purity had a lower survival rate (Figures 8C–E).

### Characteristics of Immune Cells Infiltration in Melanoma

**FIGURE 9:**
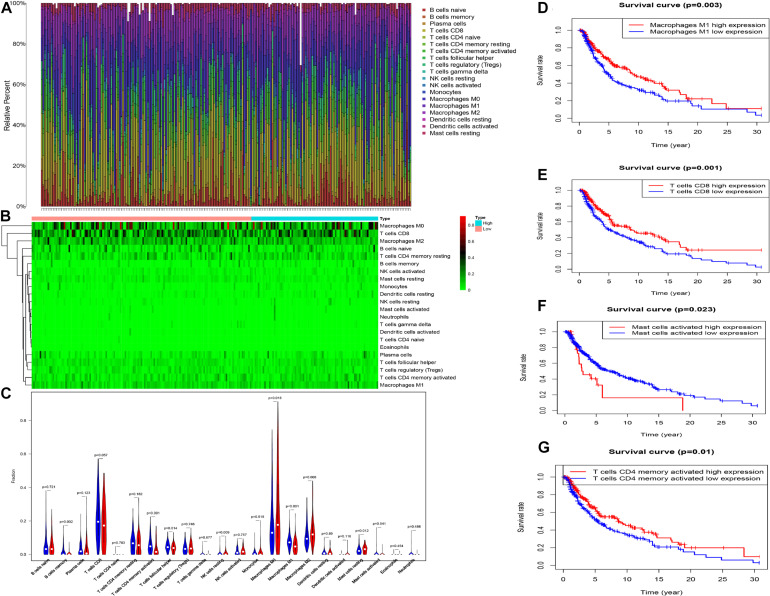
The proportion **(A)** and heat map **(B)** of 22 immune cells in whole cohort. The comparison of the composition of immune cells between two risk group (low: blue vs. high: red) **(C)**. K–M curve between high and low level of infiltrating Macrophages M1, T cells CD8, and T cells CD4 memory activated **(D–G)**.

**FIGURE 10:**
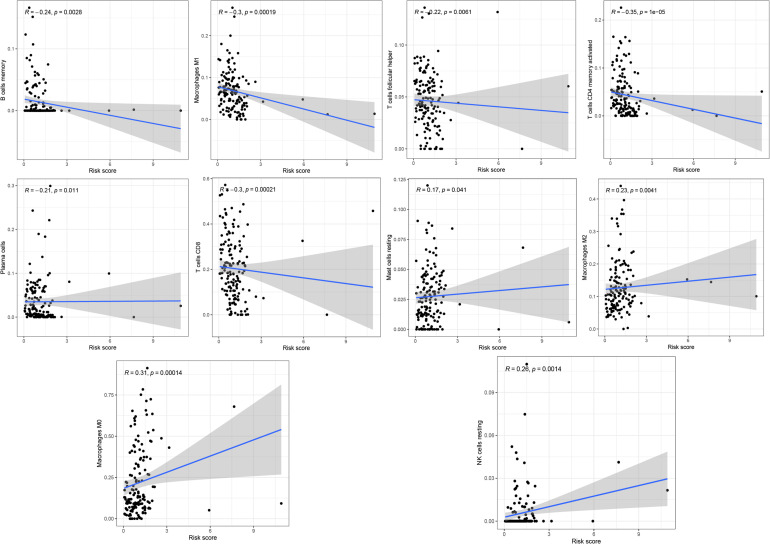
The correlation between risk score and immune cells.

The profiling of infiltrating immune cells in melanoma samples was performed using the CIBERSORT algorithm. Figures 9A,B show that Macrophages M0, CD8 T cells, naive B cells, resting memory CD4 T cells and Macrophages M2 were highly expressed in melanoma. Wilcoxon rank-sum test indicated that there were notable differences in B cells memory (*P* = 0.002), activated memory CD4 T cells (*p* < 0.001), T follicular helper cells (*p* = 0.014), resting NK cells (*p* = 0.009), Macrophages M0 (*P* = 0.010), Macrophages M1 (*P* < 0.001), Macrophages M2 (*P* = 0.008), and resting Mast cells (*P* = 0.012) between the two risk groups (high vs. low) in the whole (training and testing) set (Figure 9C). The memory B cells, activated memory CD4 T cells, T follicular helper cells and Macrophages M1were higher expressed in the low-risk group, while the resting NK cells, Macrophages M0, Macrophages M2 and resting Mast cells were higher expressed in the high-risk group (Figure 9C). Kaplan-Meier curves demonstrated that patients with high fractions of Macrophages M1, activated memory CD4 T cells, and CD8 T cells were associated with higher survival rates, while those with higher fractions of activated Mast cells are on the contrary (*p* < 0.05, Figures 9D–G). Furthermore, we explored the correlation between risk score and immune cells. The results illustrated that risk score was remarkably positively correlated with Macrophages M0 (*r* = 0.31, *p* = 0.00014), Macrophages M2 (*r* = 0.23, *p* = 0.0041), mast cells resting (*r* = 0.17, *p* = 0.041) and NK cells resting (*r* = 0.26, *p* = 0.0014), while negatively correlated with memory B cells (*r* = −0.24, *p* = 0.0028), plasma cells (*r* = −0.21, *p* = 0.011), Macrophages M0 (*r* = −0.3, *p* = 0.00019), activated memory CD4 T cells (*r* = −0.35, *p* = 1e-05), CD8 T cells (*r* = −0.3, *p* = 0.00021) and T cells follicular helper (*r* = −0.22, *p* = 0.0061, Figure 10).

## Discussion

Melanoma is one of the most aggressive cancers, associated with a worse prognosis and distant metastasis. It is important to identify new potential markers to improve the prognosis and treatment of melanomas. Numerous studies have shown that EMT plays a role in tumorigenesis, migration, metastasis, chemotherapy resistance, etc. (Mittal, 2018; Saitoh, 2018). The majority of studies have concentrated on the influence of EMT in tumor progression, resistance, and metastasis, and only a few studies have explored the prognostic value of EMT-related lncRNAs/genes in cancers, particularly in melanoma.

Here, we aimed to construct and validate a new EMT-related lncRNAs prognostic signature for melanomas. The AUC values calculated from the ROC curves in the training and testing sets showed that the prognostic signature had a strong ability for differentiating melanomas. Besides, the K–M curve, score plot, plot of survival status showed a significant relationship between the risk score and clinical features which supported the robustness of the prognostic value of our signature. Moreover, the risk score and relevant clinical features were found to be important independent prognostic factors. A nomogram was constructed to predict the prognosis of melanomas and may help to individualize treatment in melanoma patients. EMT-related lncRNA signature holds a significant prognostic value and offers a theoretical basis for EMT-related targeted treatment. Besides, GSEA results showed that most of the biological pathways were enriched in the low-risk group and that EMT plays a more regulatory role in the low-risk group. We further evaluated the melanoma samples according to their tumor microenvironment in the two risk groups using ESTIMATE and CIBERSORT algorithms.

Furthermore, among eight lncRNAs, LINC02560, AC009495.1, and AC124804.1 were unfavorable lncRNA, while AC083799.1, AC036108.3, HLA-DQB1-AS1, SPRY4-AS1, and TRG-AS1 were protective lncRNA. The majority of lncRNAs have been explored in different types of cancers. For example, LINC02560 has been demonstrated to be a potential biomarker and significantly associated with the overall survival of tongue squamous cell carcinoma of patients (Zhou et al., 2019). HLA-DQB1-AS1 acts as a potential biomarker and guides future therapy in lung adenocarcinoma (Jin et al., 2020). PRY4-AS1, which is highly expressed in tumor tissue of Laryngeal Squamous Cell Carcinoma, is an important biomarker in Laryngeal Squamous Cell Carcinoma (Gong et al., 2020). AC124804.1 shows a remarkable prognostic value in lung adenocarcinoma, where it predicts the overall survival and is associated with tumorigenesis and progression (Yu and Zhang, 2020). TRG-AS1 is high expressed in glioblastoma tissues and cells, is associated with poor prognosis, and stimulates glioblastoma cell proliferation by suppressing the expression of miR-877-5p to dysregulate the expression of SUZ12 (Xie et al., 2019). Overexpression of TRG-AS1 is associated with advanced TNM stage, lymph node metastasis, and shorter overall survival, which promotes cell proliferation, invasion, and migration of TSCC (tongue squamous cell carcinoma) by mediating the miR543/YAP1 axis (He et al., 2020). Besides, TRG-AS1 is overexpressed in hepatocellular carcinoma (HCC) cells, where it enhances HCC cell proliferation, migration, EMT, and invasion by sponging miR4500 to regulate the expression of BACH1 (Sun et al., 2020). Currently, the prognostic value and potential mechanism of three lncRNAs (AC083799.1, AC036108.3, and AC009495.1) remain unclear. Even though the prognostic performance of the eight identified lncRNAs in this study is excellent, future research exploring their potential molecular mechanism in melanoma is needed.

Previous studies have demonstrated that EMT is highly related to the tumor microenvironment (Lou et al., 2016; Jiang and Zhan, 2020), and the infiltrating immune cells are associated with patients’ survival (Jiang et al., 2018). The stromal score, immune score, ESTIMATE score, and tumor purity of melanoma samples were measured using the ESTIMATE algorithm. We revealed that the risk score was negatively correlated with the stromal score, immune score, ESTIMATE score, but positively correlated with tumor purity. Besides, high immune and ESTIMATE scores have a prognostic significance, and that the immune cell infiltration of the tumor microenvironment may have a favorable effect on the patients’ prognosis.

Furthermore, CIBERSORT was used to assess the proportions of the infiltrating immune cells. We found that activated memory CD4 memory T cells, memory B cells, T follicular helper cells, and Macrophages M1 were remarkably higher in the low-risk group while resting NK cells, Macrophages M2, Macrophages M0, and resting Mast cells were higher in the high-risk group. Besides, a high fraction of Macrophages M1, CD8 T cells, and activated memory CD4 T cells were associated with better prognosis, while a high proportion of activated Mast cells were associated with a worse prognosis. Professional phagocytes, macrophages are specialized to eliminate cellular debris and dead or dying cells and are essential parts of the tumor microenvironment (Brown et al., 2017; Heymann et al., 2019). The presence of macrophages is regarded as a negative prognostic factor in most tumor types (Ruffell et al., 2012). In this study, the fraction of macrophages M0 in the high-risk group was high. We assumed that M0 macrophages played a significant role in the development of melanoma after the transformation of monocytes. The antitumorigenic M1 phenotype promotes the immune response via the regulation of dead or dying cells and infectious agents (Biswas and Mantovani, 2010). Previous studies reveal that high infiltration of M1 macrophages is associated with a high survival rate in many tumor types (Jackute et al., 2018; Zhang et al., 2019). M2 macrophages enhance tumor invasiveness, metastasis, and angiogenesis and are related to the development of an immunosuppressive microenvironment (Brown et al., 2017; Biswas and Mantovani, 2010). Previous studies show that T follicular helper (Tfh) cells emerge in tumor-draining lymph nodes where they produce IL4 to reduce antitumor immunity and cause myeloid cells to differentiate into M2 macrophages (Shirota et al., 2017). Memory B cells play an important role in antibody-mediated responses to self- and non-self-antigens and may help in antitumor immunity. Similarly, patients in high-risk squamous cell carcinoma are reported to have a high proportion of resting mast cells and unfavorable overall survival (Che et al., 2020). The proportion of resting NK cells in the high-risk group is higher in non-small cell lung cancer (Li J. et al., 2020). CD8 T cells play an important role in tumor inhibition, through the release of cytotoxic molecules such as perforin and granzymes. Besides, CD8 T cells also produce IFNγ that enhance the expression of MHC class I antigens in tumor cells, making them better targets for CD8 T cells (Tsukumo and Yasutomo, 2018). Moreover, previous studies reveal that activated memory CD4 T cells have the least proportion in clinical stage IV of bladder cancer and are associated with beneficial prognosis, which may directly kill tumors or activate the body’s immune response to destroy the tumor cells (Li W. et al., 2020).

This study has some limitations that need to be addressed. First, this was a retrospective study that relied on public data sources. Second, the amount of data available from public databases is still limited and has not been validated using other databases. Besides, even though the studies utilized the same cohort, there may be exist the possibility of over-fitting when compared the prediction value of the EMT-related lncRNA signature with other two published lncRNA signatures. Therefore, it is important to confirm the findings through multicenter studies and actual experiments before the EMT-related lncRNA signature can be clinically used.

## Conclusion

In this study, we establish and validate an EMT-related lncRNA signature targeting EMT-related genes in melanoma, which can be clinically used to predict individualized prognosis and treatment in melanoma patients. Moreover, the EMT-related lncRNA signature is validated as an independent prognostic indicator for melanoma. This signature offers a novel tool for future clinical applications to melanoma treatment.

## Data Availability Statement

The datasets presented in this study can be found in online repositories. The names of the repository/repositories and accession number(s) can be found in the article/Supplementary Material.

## Author Contributions

BX designed the research study. BX, LL, ZC, and YZ performed the literature search and statistical analysis. BX, AL, PW, and CX interpreted the data and drafted the manuscript. BX, HL, and TX critically revised the manuscript. All authors read and approved the final manuscript.

## Conflict of Interest

The authors declare that the research was conducted in the absence of any commercial or financial relationships that could be construed as a potential conflict of interest.

## References

[B1] Bakir B. Chiarella A. M. Pitarresi J. R. Rustgi A. K. (2020). EMT, MET, plasticity, and tumor metastasis. *Trends Cell Biol.* 30 764–776. 10.1016/j.tcb.2020.07.003 32800658 PMC7647095

[B2] Biswas S. K. Mantovani A. (2010). Macrophage plasticity and interaction with lymphocyte subsets: cancer as a paradigm. *Nat. Immunol.* 11 889–896. 10.1038/ni.1937 20856220

[B3] Bommareddy P. K. Silk A. W. Kaufman H. L. (2017). Intratumoral approaches for the treatment of melanoma. *Cancer J.* 23 40–47. 10.1097/PPO.0000000000000234 28114253

[B4] Boone B. Van Gele M. Lambert J. Haspeslagh M. Brochez L. (2009). The role of RhoC in growth and metastatic capacity of melanoma. *J. Cutan. Pathol.* 36 629–636. 10.1111/j.1600-0560.2008.01117.x 19222696

[B5] Brabletz T. (2012). To differentiate or not–routes towards metastasis. *Nat. Rev. Cancer* 12 425–436. 10.1038/nrc3265 22576165

[B6] Brown J. M. Recht L. Strober S. (2017). The promise of targeting macrophages in cancer therapy. *Clin. Cancer Res.* 23 3241–3250. 10.1158/1078-0432.CCR-16-3122 28341752 PMC5529121

[B7] Che Y. Luo Z. Zhang C. Sun N. Gao S. He J. (2020). Immune signature of tumor-infiltrating immune cells predicts the prognosis and therapeutic effects in squamous cell carcinoma. *Int. Immunopharmacol.* 87:106802. 10.1016/j.intimp.2020.106802 32745903

[B8] De Craene B. Berx G. (2013). Regulatory networks defining EMT during cancer initiation and progression. *Nat. Rev. Cancer* 13 97–110. 10.1038/nrc3447 23344542

[B9] Gong S. Xu M. Zhang Y. Shan Y. Zhang H. (2020). The prognostic signature and potential target genes of six long Non-coding RNA in laryngeal squamous cell carcinoma. *Front. Genet.* 11:413. 10.3389/fgene.2020.00413 32411183 PMC7198905

[B10] He S. Wang X. Zhang J. Zhou F. Li L. Han X. (2020). TRG-AS1 is a potent driver of oncogenicity of tongue squamous cell carcinoma through microRNA-543/Yes-associated protein 1 axis regulation. *Cell Cycle* 19 1969–1982. 10.1080/15384101.2020.1786622 32615889 PMC7469544

[B11] Heymann M. F. Lezot F. Heymann D. (2019). The contribution of immune infiltrates and the local microenvironment in the pathogenesis of osteosarcoma. *Cell Immunol.* 343:103711. 10.1016/j.cellimm.2017.10.011 29117898

[B12] Jackute J. Zemaitis M. Pranys D. Sitkauskiene B. Miliauskas S. Vaitkiene S. (2018). Distribution of M1 and M2 macrophages in tumor islets and stroma in relation to prognosis of non-small cell lung cancer. *BMC Immunol.* 19:3. 10.1186/s12865-018-0241-4 29361917 PMC5781310

[B13] Jiang Y. Zhan H. (2020). Communication between EMT and PD-L1 signaling: new insights into tumor immune evasion. *Cancer Lett.* 468 72–81. 10.1016/j.canlet.2019.10.013 31605776

[B14] Jiang Y. Zhang Q. Hu Y. Li T. Yu J. Zhao L. (2018). ImmunoScore signature: a prognostic and predictive tool in gastric cancer. *Ann. Surg.* 267 504–513. 10.1097/SLA.0000000000002116 28002059

[B15] Jin D. Song Y. Chen Y. Zhang P. (2020). Identification of a Seven-lncRNA immune risk signature and construction of a predictive nomogram for lung adenocarcinoma. *Biomed. Res. Int.* 2020:7929132. 10.1155/2020/7929132 32596372 PMC7273488

[B16] Li C. H. Chen Y. (2013). Targeting long non-coding RNAs in cancers: progress and prospects. *Int. J. Biochem. Cell Biol.* 45 1895–1910. 10.1016/j.biocel.2013.05.030 23748105

[B17] Li J. Li X. Zhang C. Zhang C. Wang H. (2020). A signature of tumor immune microenvironment genes associated with the prognosis of nonsmall cell lung cancer. *Oncol. Rep.* 43 795–806. 10.3892/or.2020.7464 32020214

[B18] Li W. Zeng J. Luo B. Mao Y. Liang Y. Zhao W. (2020). [High expression of activated CD4(+) memory T cells and CD8(+) T cells and low expression of M0 macrophage are associated with better clinical prognosis in bladder cancer patients]. *Xi Bao Yu Fen Zi Mian Yi Xue Za Zhi* 36 97–103. 32314705

[B19] Lou Y. Diao L. Cuentas E. R. Denning W. L. Chen L. Fan Y. H. (2016). Epithelial-mesenchymal transition is associated with a distinct tumor microenvironment including elevation of inflammatory signals and multiple immune checkpoints in lung adenocarcinoma. *Clin. Cancer Res.* 22 3630–3642. 10.1158/1078-0432.CCR-15-1434 26851185 PMC4947453

[B20] Mishra H. Mishra P. K. Ekielski A. Jaggi M. Iqbal Z. Talegaonkar S. (2018). Melanoma treatment: from conventional to nanotechnology. *J. Cancer Res. Clin. Oncol.* 144 2283–2302. 10.1007/s00432-018-2726-1 30094536 PMC11813321

[B21] Mittal V. (2018). Epithelial mesenchymal transition in tumor metastasis. *Annu. Rev. Pathol.* 13 395–412. 10.1146/annurev-pathol-020117-043854 29414248

[B22] Newman A. M. Liu C. L. Green M. R. Gentles A. J. Feng W. Xu Y. (2015). Robust enumeration of cell subsets from tissue expression profiles. *Nat. Methods* 12 453–457. 10.1038/nmeth.3337 25822800 PMC4739640

[B23] Nieto M. A. Huang R. Y. Jackson R. A. Thiery J. P. (2016). EMT: 2016. *Cell* 166 21–45. 10.1016/j.cell.2016.06.028 27368099

[B24] Nilchian A. Johansson J. Ghalali A. Asanin S. T. Santiago A. Rosencrantz O. (2019). CXADR-mediated formation of an AKT inhibitory signalosome at tight junctions controls epithelial-mesenchymal plasticity in breast cancer. *Cancer Res.* 79 47–60. 10.1158/0008-5472.CAN-18-1742 30385615

[B25] Pastushenko I. Blanpain C. (2019). EMT transition states during tumor progression and metastasis. *Trends Cell Biol.* 29 212–226. 10.1016/j.tcb.2018.12.001 30594349

[B26] Ruffell B. Affara N. I. Coussens L. M. (2012). Differential macrophage programming in the tumor microenvironment. *Trends Immunol.* 33 119–126. 10.1016/j.it.2011.12.001 22277903 PMC3294003

[B27] Saitoh M. (2018). Involvement of partial EMT in cancer progression. *J. Biochem.* 164 257–264. 10.1093/jb/mvy047 29726955

[B28] Shirota H. Klinman D. M. Ito S. E. Ito H. Kubo M. Ishioka C. (2017). IL4 from T follicular helper cells downregulates antitumor immunity. *Cancer Immunol. Res.* 5 61–71. 10.1158/2326-6066.CIR-16-0113 27920023 PMC6385600

[B29] Sun X. Qian Y. Wang X. Cao R. Zhang J. Chen W. (2020). LncRNA TRG-AS1 stimulates hepatocellular carcinoma progression by sponging miR-4500 to modulate BACH1. *Cancer Cell Int.* 20:367. 10.1186/s12935-020-01440-3 32774161 PMC7401190

[B30] Tsukumo S. I. Yasutomo K. (2018). Regulation of CD8(+) T cells and antitumor immunity by notch signaling. *Front. Immunol.* 9:101. 10.3389/fimmu.2018.00101 29441071 PMC5797591

[B31] Wenandy L. Sorensen R. B. Svane I. M. Thor Straten P. Andersen M. H. (2008). RhoC a new target for therapeutic vaccination against metastatic cancer. *Cancer Immunol. Immunother.* 57 1871–1878. 10.1007/s00262-008-0517-2 18415097 PMC11030672

[B32] Wu L. Zhu L. Li Y. Zheng Z. Lin X. Yang C. (2020). LncRNA MEG3 promotes melanoma growth, metastasis and formation through modulating miR-21/E-cadherin axis. *Cancer Cell Int.* 20:12. 10.1186/s12935-019-1087-4 31938020 PMC6954595

[B33] Xie H. Shi S. Chen Q. Chen Z. (2019). LncRNA TRG-AS1 promotes glioblastoma cell proliferation by competitively binding with miR-877-5p to regulate SUZ12 expression. *Pathol. Res. Pract.* 215:152476. 10.1016/j.prp.2019.152476 31196742

[B34] Yoshihara K. Shahmoradgoli M. Martinez E. Vegesna R. Kim H. Torres-Garcia W. (2013). Inferring tumour purity and stromal and immune cell admixture from expression data. *Nat. Commun.* 4:2612. 10.1038/ncomms3612 24113773 PMC3826632

[B35] Yu X. Zhang Y. (2020). Identification of a long non-coding RNA signature for predicting prognosis and biomarkers in lung adenocarcinoma. *Oncol. Lett.* 19 2793–2800. 10.3892/ol.2020.11400 32218832 PMC7068299

[B36] Zhang S. Zhang E. Long J. Hu Z. Peng J. Liu L. (2019). Immune infiltration in renal cell carcinoma. *Cancer Sci.* 110 1564–1572. 10.1111/cas.13996 30861269 PMC6501001

[B37] Zhou R. S. Zhang E. X. Sun Q. F. Ye Z. J. Liu J. W. Zhou D. H. (2019). Integrated analysis of lncRNA-miRNA-mRNA ceRNA network in squamous cell carcinoma of tongue. *BMC Cancer* 19:779. 10.1186/s12885-019-5983-8 31391008 PMC6686570

